# Embodying Experiences with Nature in Everyday Life Recovery for Persons with Eating Disorders

**DOI:** 10.3390/ijerph17082784

**Published:** 2020-04-17

**Authors:** Lise Katrine Jepsen Trangsrud, Marit Borg, Solfrid Bratland-Sanda, Trude Klevan

**Affiliations:** 1 Department of Health, Social and Welfare Studies, University of South-Eastern Norway, Postboks 235, 3603 Kongsberg, Norway; marit.borg@usn.no (M.B.); trude.goril.klevan@usn.no (T.K.); 2 Department of Sport, Physical Education and Outdoor Studies, University of South-Eastern Norway, Postboks 235, 3603 Kongsberg, Norway; solfrid.bratland-sanda@usn.no

**Keywords:** *Friluftsliv*, recovery, health promotion, nature, everyday life, eating disorders, hermeneutic phenomenology

## Abstract

Eating disorders can be understood as attempts to manage a problematic relationship with one’s own body. The objective of this qualitative study was to explore and discuss perspectives of embodying “experiences with nature” related to recovery in everyday life for persons experiencing eating disorders. The study was carried out in the context of a hermeneutic phenomenological approach. Eight participants with an interest in nature and *friluftsliv* (outdoor pursuits), and with experiences with bulimia nervosa and/or binge-eating disorders, were interviewed twice. Interviews took place in nature, in combination with a “going together” method. The results reveal how the participants highlighted experiences with nature as accentuating feelings of calmness and an engagement of the senses. Participants described nature as a non-judgmental environment that also provided room for self-care. This article explores the implications of everyday life perspectives on nature in recovery, as well as of an integrated focus on body and mind in experiences with eating disorders. The article concludes with an emphasis on how participant’s embodying experiences with nature enabled a (re)connection with one’s own body.

## 1. Introduction

In recent decades, there has been growing concern around an expanding human dislocation and alienation from nature [1,2,3]. The increasing technification and urbanization of human life have literally paved the road for lifestyles requiring less physical activity, including less in-person contact among humans and between humans and nature [2,4]. This concern has been forwarded in parallel with the increasing amount of international research supporting the hypothesis that interaction with nature holds multiple beneficial effects for health [5]. Related to this, nature has been deemed vital in both health promotion and mental health recovery [6]. However, while this research often focuses upon the psychological, physiological, social, and spiritual effects of nature [5,6], there is limited research with an explicit focus on embodying “experiences with nature” [7,8].

Understandings of embodying experiences are frequently connected to the “bodily turn” of phenomenology, which represents an effort to turn away from the Cartesian dualism between mind and body [9,10]. As famously argued by Merleau-Ponty [10], our body is not only something we have, it is also what we are. As such, the understanding that to be a subject is to be in the world as a body [11] holds several ontological and epistemological implications. If we are our body, then we must know that all that we have is acquired through our body: We live, perceive, and sense the world with our body and all our previous experiences, our background, and social relations are incorporated within our body [12]. Thus, “embodiment emphasizes the body as a psychological, cultural and historical phenomenon as well as a biological and material one” [12] (p. 80).

The understanding that we experience the world through our body and through our senses holds several consequences for how we understand persons’ experiences in eating disorder (ED) recovery. Especially pertinent might be the emphasis placed on the idea that the body is our “means of communication” with the world [11] (p. 92). Eating disorders (EDs) are defined as preoccupations with food, body shape, and weight [13] and may be argued as embodied ways of communicating with the world, as well as efforts to manage a problematic relationship with one’s own body, thoughts, and feelings [14,15]. EDs are still heavily taboo; bulimia nervosa (BN) and binge eating disorder (BED), in particular, are related to features perceived as negatively loaded—loss of control, overeating, binging, and vomiting—and often leading to shame and guilt [16]. In accordance with literature arguing against the idea that anorexia nervosa (AN) constitutes a “blanket term” for all EDs [17], this study seeks to broaden the understanding that EDs are multifaceted by accentuating the stories of persons experiencing BN and/or BED.

Although there has been a growing trend towards integrating a focus on the body in recovery for persons experiencing EDs, this approach has largely been conceptualized as an issue related to a disturbed body image or body perception [18]. While this could be seen as a clinical approach towards recovery, often including assessment of outcomes such as “body dissatisfaction”, one could argue that our relationship with our own body is diverse and too complex to be reduced to individual concepts [14]. Contrasting the clinical perspective on recovery *from* EDs is a focus on recovery as personal, social, and contextual processes [19,20], as well as embodied experiences lived *in* everyday life. Thus, recovery is not typically seen as an outcome, nor as a linear process; rather, the emphasis on the everyday perspective in recovery argues that most of our health and wellbeing is maintained in our daily life through our coping strategies and through what we deem meaningful [21,22]. Closely related to this is the perspective that recovery should be seen as ongoing processes, in which aspects of connectedness, hope, finding identity and meaning beyond the challenges or illness experienced, and empowerment (given the acronym CHIME) has been emphasized [23]. As research has been inconclusive in terms of effective strategies for treatment of EDs [24], including ongoing discussions with regards to what *ED recovery* actually implies [25], there is a need for a focus on everyday situations as it emphasizes how persons can find ways to live with the situation while seeking to ameliorate the negative effects [19,21].

The objectification of the body that appears prominent in much of the literature related to EDs [14] can be contrasted with the subjective and embodied experiences seen as essential in the human–nature interaction [26]. The present study was situated in a Norwegian context, where *friluftsliv* (often related to “outdoor pursuits” or “outdoor life”), understood as comprising nature-based, non-competitive, and non-motorized activities (either sedentary or active) [27], constitutes the cultural context in which the experiences with nature take place. In the Nordic countries, *friluftsliv* is emphasized as an arena that can facilitate support, engagement, and coping strategies on an everyday basis, and as an important source of health and wellbeing [5,28].

However, while the contextual situatedness of the body proves important [10], one could argue that experiences with nature are also subjective, and that the ways in which we experience nature are influenced by social and cultural relations [29]. Related to this, it is important to note that *friluftsliv* is subject to a range of understandings and constructions, and is a dynamic and multifaceted phenomenon [30]. The understanding of *nature* in the current study is also broad, in the sense that it is defined by the ways in which the participants related to it. Situated in the Norwegian landscape, nature included forests close to urban cities and more remote wilderness areas.

In accordance with the shift from a focus on the mind towards the body—and, recently, an integrative focus on body *and* mind—in experiences with ED recovery, nature has been emphasized as holding great potential [27,31]. In a recent study of nature-based therapy for persons with BED, nature was reported to facilitate several positive processes, including increased wellbeing and enhanced self-esteem [31]. This remains a relatively unexplored field, however. As such, this project aimed to contribute to a limited area of research, particularly research attending to first-hand experiences with ED recovery [32,33,34]. Following this, the objective of this article is to explore embodying experiences with nature related to recovery in everyday life for persons experiencing EDs.

## 2. Methods

In keeping with its aim, this study was carried out in the context of a hermeneutic phenomenological approach. This approach allows for exploring and developing an in-depth understanding and new insights into human life [35], while also acknowledging that studies of a phenomenon are always interpreted and contingent [36]. The present study was based on the epistemological assumption that meaning occurs and knowledge is developed within a context in which the participant, the researcher, and the research project including the environmental context, as a whole take part [37,38]. The dialogical perspective of Gadamer [39] in his “fusion of horizons” provides a methodological approach that fits well with an explorative and interpretative qualitative study, allowing the researcher to actively engage with the participants’ experiences, albeit in a thoroughly self-critical, contextualized, and reflexive manner [37]. In order to maintain transparency in the combination of phenomenological exploration and hermeneutic reflection, the research project must have a clearly outlined structure, and the first author must maintain a practice of reflexive writing at each stage of the research process [37,40].

### 2.1. User-Involvement in the Research Process

The current project was developed in collaboration with a Norwegian user organization focusing on eating disorder experiences (*Rådgivning om spiserforstyrrelser*—ROS), to ensure that the first-person perspective was included in the research process. Furthermore, a “competence group” [21,41] consisting of between five and seven persons with experiences in mental health challenges and in EDs, and with interests in nature and *friluftsliv*, were invited to participate in the development and analysis of the study. The group contributed to the formation of key themes for the interviews and the development of the methodological approach; they also provided advice throughout the project, based on their experiences and perspectives. Some of the participants had taken the University of South-Eastern Norway’s post-graduate course in user-involved collaborative research in mental health. Albeit having been criticized for resulting in “tokenism” [42], user-involvement in research entails a recognition of the knowledge of those who are “experts by experience” [43]. While the political-ideological contribution of implementing user-involvement can be called into question, we find it important to highlight the use-value of forming a competence group. In the current project, the competence group took on more of an advisory function in the different stages of the research [43]. The group acted as a safeguard to ensure that our focus remained centered on the participants and their experiences with nature, and to remind us of the complexity of everyday situations to avoid over-simplification of our interpretations of the data [44].

### 2.2. Recruitment Procedures

Participants were recruited during the summer of 2017 through posts on ROS’s website. In addition, the special health services for treatment related to EDs in Norway were contacted, and flyers were posted at several universities in Eastern Norway (including in the student health services). In line with the objective of the study, the following inclusion criteria were applied:

- Interested in nature and *friluftsliv*
- Self-reported experiences with bulimia nervosa and/or binge eating disorder
- Above 18 years of age
- Both men and women

Participants were excluded if currently residing in institutions, or facing somatic health problems.

### 2.3. Participants

Upon reading the recruitment text, eight persons contacted the first author during the summer of 2017, and volunteered to participate in the research project. This included seven women and one man between 19 and 41 years of age. The participants came from across Norway, although most of them were currently living in the southern part of the country. Two came from other Nordic countries, but were currently living in Norway. They were all studying and/or working—none were unemployed. All were either currently enrolled in or had previously completed some degree of higher education. The participants demonstrated great variety in terms of their previous experiences with nature and friluftsliv. However, all of the participants informed us that the value of such experiences was their main motivation for participation in the research project. Their experiences with EDs ranged from 2 to 25 years. Although some declared that things were better now than previously, they related that they were still living with the consequences of the EDs. Two of the participants stated that they currently received help from professionals; six said they had a supportive social network of friends and family.

In the research process, we relied on the participants’ self-reported experiences with EDs and no medical records were obtained. Although the study’s focus was on persons with experiences from the above-mentioned diagnoses, EDs are a complex phenomenon and several of the participants expressed that they could see themselves fit into more than one of the diagnostic categories. Related to this, some of the participants described previous experiences with other types of EDs, as well—for example, AN in addition to BN/BED. The number of participants included in the study were continually assessed in terms of “information power.” This meant an evaluation of the quality of the data material with regard to the aim of the study, the specificity of the participants’ experiences, the availability of established theoretical perspectives, the quality of the dialogue between researcher and participants, and the analytical strategy [45].

### 2.4. Data Creation

This study consisted of two individual meetings with each participant (one person was only met with once, due to their health situation). The first meeting consisted of an unstructured walking interview (hereafter termed “going together”) in nature. The second meeting was a sit-down, semi-structured interview.

#### 2.4.1. Going Together

Having established contact, the participants were asked to decide upon a place in nature to meet. Although the meeting was not specified as either sedentary or active, all of the participants invited the first researcher for a walk in a natural area near them. Information describing the purpose of and procedure for the study was sent to the participants prior to the first meeting; in addition, information was provided upon meeting. Having obtained informed and written consent, both the participant and the first author were equipped with a small microphone and a recorder and the conversation that took place during the walk was audio-recorded. The topics initiated by the first author during the unstructured interview were intended to elicit participants’ reflections on why we were meeting in this particular place, and how they experienced nature as we were walking. The walks lasted between 60 and 90 min; four took place in the forest, two around a lake, one in the mountains, and one along a river.

The method chosen has parallels with methods used by other researchers on *friluftsliv* [29,46,47]. This method shares similarities with go-along methods used in other countries [48,49], in that “going together” enabled exploration of the interaction between the participant and the environment, and aspects of lived experiences as contextual [49]. Allowing the data creation to take place in nature provided the first author with the opportunity to observe participants’ body language while also paying attention to the conversation.

#### 2.4.2. Interviews

The “sit-down”, semi-structured, open-ended qualitative interview took place approximately two weeks after the first meeting. This approach was selected for further exploration of the person’s experiences and perspectives. The transcript, preliminary analysis and first author’s fieldnotes from the first meeting constituted the point of departure for the interview. The second meeting, also recorded, followed a semi-structured interview guide focusing upon the meaning of nature and *friluftsliv* in participants’ everyday life. The questions were related to participants’ stories of how they engaged with nature in their everyday life; for instance, where they preferred to be and what they liked to do, how they related to nature and why they believed they became interested in spending time outdoors. Moreover, stories about childhood experiences with nature as well as experiences with nature as both supportive and/or challenging, were important topics during the conversation. During the first meeting, the participants expressed a preference for the sit-down interview to be conducted outdoors. With two exceptions, due to practical circumstances, the sit-down interviews therefore took place in a natural area near them: in a shelter in the forest, on a bench along a fjord (two occasions), on a blanket beside the river, and in a park. The participant and the first author were the only ones present at both meetings.

### 2.5. Data Analysis

The method of analysis was interpretative phenomenological analysis (IPA) [50]. While IPA has been argued primarily as a psychological method [51], it can be seen as being of value in other disciplines, as well [50]. The IPA method is largely based on the hermeneutic circle as an iterative and inductive process, in which the researcher moves from the ideographic to the shared and back to the nuances [50]. The data were analyzed by merging IPA with the computer software program NVivo [52].

The initial phase of the IPA approach included listening to the recordings several times. Any thoughts regarding the interview setting or the text were written down in a separate document and saved [50]. The recordings were also transcribed verbatim in NVivo [52]. The second phase involved preliminary note-taking related to the first author’s understanding of how the participant talked about, understood, and related to nature and *friluftsliv*. This included descriptive, linguistic (where appropriate), and interpretative comments. The comments were then assigned different colors in NVivo [50]. During the third phase, emergent themes (or “nodes,” in NVivo), based on the first author’s preliminary notes were developed [50]. During this phase, the goal was to keep as much of the language used by the participants as possible, to ensure a proximity to the empirical material in the coding process. The fourth phase included looking for connections across the themes [50]; the emergent themes were then organized into hierarchical groups of nodes. The fifth phase consisted of moving to the next participant and repeating the process. The sixth phase of the analysis included looking for patterns across all the themes, across all the participants, and organizing the nodes into themes and sub themes [50]. In reporting the results, a prominent task was to present the nuances in the participants’ experiences, in keeping with the ideographic emphasis of the IPA [50].

The analysis of the transcripts was reviewed together with the initial fieldnotes and observational comments were written directly after each meeting. The direct quotes from the participants were translated by the first author for this article, but were kept in their original language until the article was proofread. Although the co-authors were thoroughly involved in all stages of the research, the first author carries the main responsibility for the analysis and presentation of the results.

### 2.6. Ethical Considerations

The project was assessed and approved by the Norwegian Centre for Research Data and the Regional Committees for Medical and Health Research Ethics (2017/519C). However, as argued by Tee and Lathlean [53], ethical guidelines cannot account for the complexities in specific situations. Hence, it became important to view information and consent as a process rather than an absolute. The notion of “ethical mindfulness” as everyday ethics was important throughout the research process—this included attention to the relationship between researcher and participant [54] and a continuing assessment of potentially vulnerable situations [53].

A few of the participants commented that it was demanding to be walking, paying attention to nature, *and* taking part in an interview at the same time. They expressed a wish for extended time related to the first meeting. Consequently, the recruitment text and information provided to participants were updated to include the fact that time limits were unspecified. However, the meetings that followed tended to be a similar length as those that took place before the update. As many of the topics for the sit-down interview were often touched upon during the first meeting, the necessity of the second meeting was discussed after interviews with three participants. It was decided that the second meeting should be kept, due to the variation between the “going together” dialogue and that in the interview. Moreover, it proved valuable to have the participants reflecting upon our first meeting. Data were made anonymous through the transcribing process via moderating or removing details that could allow the identification of participants. All the participants in the study were given pseudonyms.

## 3. Results

The results of the current study emphasize the participants’ embodying experiences with nature in everyday life. The results reveal how nature provided participants with experiences of peacefulness and calmness, and facilitated re-embodying experiences through the engagement of their senses—particularly the sensing of the ground with their feet. Moreover, nature had room to embrace their whole body, complete with their experiences, and this opened up an understanding of it being legitimate, and even required, that they take care of themselves in nature. Each of these themes are introduced with an observational reflective note related to the context, marked in italics. Following the observational comment, we have included a short summary of the sub-themes to be explored within each of the sections.

### 3.1. Nature Provides One with Peacefulness and Calmness

*We had been walking for some time in a forest near the city. Although we were surrounded by the trees, once in a while we heard cars passing from the nearby streets. After a while, having moved further away from the streets, there was no traffic to be heard and we stopped in front of an ice-covered, white area. “Aaaah,” Jakob breathed in, and then out, lowering his shoulders. “This is the lake!”*

All of the participants talked about how nature provided them with peacefulness and calmness, albeit in multiple ways. The sub themes to be explored in the following are related to the calming qualities of nature, such as wide-open spaces and quietness, as well as highlighting how the participants’ experiences with nature provided them with calmness, through an engagement of their senses and by a sense of belonging in the greater connection of nature.

Several of the participants expressed how much of the distress related to the ED involved a feeling of a pressure building up inside, and they just had to get out. Once in nature, they explained, their heartbeat would slow down, and the open spaces made it easier to breathe. Moreover, the participants described how the peacefulness and calmness they found in nature allowed for an engagement of their senses. Closely related to this, they highlighted the importance of the quietness in nature—not necessarily a total quietness, but one in which “the city sounds,” as Julie called them, were absent. They emphasized that many of the features they were influenced by in society were gone in nature, or were experienced differently and as less demanding. As we were walking up a small hill in the forest, Nora described how she became aware that she was repeatedly touching the grass and she highlighted how sensing through her fingers elicited a calmness in her. She continued:

But it’s like, using nature and using *friluftsliv* has become a thing for me, in order to deal with it. I have, well, many people try mindfulness and stuff, I can’t…I couldn’t do that. Sitting there and searching for things you don’t want to feel. Sitting still and sensing your body even more, I can’t do that. So my kind of mindfulness is out here, where we are now, to change one’s focus… sensing nature, listening to the birds, the smell, and it’s like, it calms me down, in a good way. I believe this is the most important reason that I manage my life as well as I do.

Like several of the participants, Nora expressed how touching, listening, and smelling nature were essential to her, and she related the calmness she experienced in the sensible relationship with nature to a shift in focus, from herself towards nature. This change of focus onto something more than oneself seemed to be important and connected to an experience of bodily calmness for several of the participants. With nearly all of the participants, I found that they typically stopped when we walked out of the forest and came upon a view across the valley, towards the mountains, or when the view opened up towards a lake or a river. The participants described a feeling of calmness, for instance by looking at the mountains. Related to this, Kristine commented: “Every morning when I go to work, or just go downtown, I look towards the mountains, and I believe it fills me with calmness”. As was highlighted by several of the participants, she continued to explain: “It becomes kind of possible to lift your perspective and it’s like a higher truth than those things you normally think about in your daily life.” In comparison to their descriptions of what they felt to be a relatively shifting and unstable society, the mountains, the trees, and the rocks represented constancy. In line with this, Maren reflected:

How long has that rock existed? How long has that tree been here? How old is it, approximately? I often wonder about things like that. Because I find it so fascinating. This rock, it has witnessed this and that, from hundreds of years ago, or even millions of years ago. So, in the bigger picture, my life and my problems are perhaps not that big after all.

Several of the participants described how they experienced a peacefulness in nature related to a sense of belonging. They expressed an understanding that there was something inherent in humans’ innate relationship with nature, and how they were influenced by nature’s own balance. As described by Maren in the above quotation, seeing themselves as part of a greater connection also implied that they could give their own troubles a little less attention. The majority of the participants recalled how much of their effort was expended on alleviating some of the distress related to the ED. Related to this, nature was described as a calm, quiet and stable place, and this outer calmness seemed to influence the participants by filling them with calmness on the inside.

### 3.2. Nature Invites One to Sense the World with One’s Feet

*We had been walking in silence for a while on our way down through the forest. The path she had chosen for us to walk wound between the trees. It was narrow, covered with needles and warm after the sun had been heating it all day. Then Nora commented: “This is such lovely forest ground to walk on… with the spruce needles; it becomes so soft and nice…”*

A prominent theme across all the participants’ stories were their reflections upon sensing the ground with their feet. The sub themes in the following section are related to how the qualities of the ground required one’s presence and how this focus “down” was experienced as re-embodying.

While we walked in nature, several of the participants emphasized the characteristics and qualities of the ground. It was especially the softness of a path covered with needles from spruce or pine trees and the sensing of one’s feet in relation to the ground that was highlighted. Maren put it this way:

But then, at least we walked on a *path*. I prefer that. When it is soft to walk on and there’s like little rocks and streams and needles from the spruces, or maybe pines in this case [she laughs], needles on the forest ground, it makes it nice to walk there. It is something about the feeling of sinking a bit into the ground.

As with Maren, several of the participants also highlighted how rocks and roots were experienced as natural features that captured their attention. This meant, they explained, that they had to be present and attentive to where they placed their feet. In comparison, asphalt or gravel presented little variation. Most of the participants distinguished clearly between the feelings elicited in their body from walking on asphalt or gravel and those from walking on a “natural” path. In addition, several of the participants highlighted how the variation in the terrain meant that time and distance were felt differently.

These reflections regarding sensing nature with one’s feet became pertinent, as several of the participants talked about their ED as something going on in their head and in the upper part of their body: Nora described it as having ants in her head; Vanessa described it as having someone sitting on her back constantly telling her what to do or how to feel; and Eva related to her ED as a troubling “voice” creating troubling thoughts. In addition, though the EDs were mostly described by participants as being connected to troubling thoughts or feelings located in their head, the EDs were also described as numbing their body, as if they were shutting down contact with the rest of their body.

Kristine: It’s like, eating disorders are all about cutting off here, by the neck [pointing to her neck], and you are only present in your head. So it’s almost like, your body is disappearing.

Kristine related that she experienced having an ED as if her body was gone. Being in nature, she continued, became important, as it was a way of “re-embodying” herself by “getting a break and getting down into [her] body.” Although most of the participants emphasized their devotion for natural paths, they also gave examples of times when the distress related to the ED became so stressful that they did not care about their environment anymore. Thus, while they highlighted a preference for nature with as little human impact as possible, in their daily life, they often made use of the nearby forest. However, when things became too difficult, they would go for a run wherever they could, though still preferably in city parks.

### 3.3. Nature Embraces One As One Is

*While we were walking, we moved in and out of our conversation. Sometimes just walking in our own thoughts, and observing and being in nature. With all of the participants, I experienced situations when things happened around us, like when a squirrel appearing, a duckling, a flower, the rain, or a view caught our attention and became integrated into the conversation. The fluctuations of our attention seemed closely connected to the participants’ emphasis on nature as places where they could get a break from their troubling thoughts and just be as they are.*

The participants talked about feeling as though nature welcomed them the way they are. The sub themes to be explored in the following section are related to the participants’ expressions of nature as being non-judgemental, as providing a break from the ED related distress as well as providing room for whatever they brought with them, inclusive of troubling thoughts and feelings.

In nature, the participants explained, no one was watching them as when there were many people around. Most of the participants ventured into nature alone, as well as together with friends and family. Still, the experience of not being judged by anyone while in nature was emphasized, and the participants described a feeling of being left in peace. Related to this, Kristine commented that being in nature provided a break from the constant, critical self-evaluation she recalled experiencing when other people were around. The feeling that one is not “wrong” in nature, which several of the participants emphasized, implies that, in nature, they experienced feeling that they were good enough, just as they are.

Kristine: One of the fundamental feelings for most people, like the low self-worth you find among persons with eating disorders, it’s this feeling that there is something basically wrong with you, that you are not like everybody else. But, when I am in nature, alone or together with others, I don’t have that feeling, because we are all natural.

The participants often described being in nature as a way of getting a break from everything going on in their mind. Not everyone expressed a desire to actually feel their body, but most of the participants emphasized that they would like to just “be” and get a break from the ED-related distress. All of the participants in this study related examples of how nature captured their attention, thereby shifting their focus: for instance, when they explored new paths and found new connections, got lost and had to find their way back, oriented themselves in the fog, or found a way to cross a river. Several of the participants emphasized how nature could be understood as providing a break from troubling thoughts through a continuous shifting between walking with an internal focus on their own thoughts and walking while focused externally on nature.

Eva: It’s nice to be active and sort of entering a monotonic, just walking, and disappearing a bit into it in a way, and sort of being present in nature and not thinking about anything else.

While nature was described as providing a break from the experienced distress in their situations, the participants also related how they experienced nature as places where they could find room to express difficult feelings. Several of them recalled episodes in which they had shouted in the wind and the rain, or at the waves and the ocean.

Vanessa: No, it’s just kind of liberating, I don’t know, it’s like, even if I had, especially one time I recall thinking as I was walking along the shore and it was blowing and raining and the waves were crashing and it was just… and I had a shitty day, but it was just, ahh, amazing.

However, while the participants described their troubles as receiving less attention as their senses were oriented more towards nature, they explained that it was not necessarily that everything difficult disappeared; instead, as Vanessa highlighted above, it was more that nature had space for all that they carried with them. Nora put it this way: “Because all the thoughts and the distress, this inner distress I have... it’s there, all the time, it never disappears, I just have to find ways to live with it, if I, in order to have a good life…” Being in nature, the participants expressed, was one way of dealing with all that was difficult, as nature was described as a place that embraced all of a person, without judging.

### 3.4. Nature Provides Room for Self-Care

*Meeting all the participants in nature meant that we had to dress according to weather conditions and natural terrain. As it turned out, this created room for valuable reflections upon how nature opened up for an awareness of one’s own bodily needs.*

According to the participants’ expressions of nature as providing room for self-care, the sub themes to be explored in the following are related to how the conditions in nature were requiring one to team up with one’s body. Moreover, as covering one’s basic needs became the most important in nature, the participants expressed how this pursued a shift from bodily appearance to bodily function.

Although most of the participants emphasized that nature had room for whatever they brought with them, they also highlighted that they experienced nature as requiring something of them. They described how nature provided direct responses and consequences, which meant that they had to listen both to nature and to their body.

Anna: And, one of the things I think about right now, which occupies my mind, is that, damn, I am about to leave on a trip and I am worried I will be eating too much because my body will be hungry. And, I am afraid I will eat too little so that I will start freezing and that I will not have enough energy to participate and be attentive. So it’s kind of an balancing act, where nature sort of requests you to… ‘In order to be *in* me, in order to use me, you have to take care of yourself.’

As Anna highlighted, nature puts the ED on trial; indeed, in order to have a positive experience in nature, several of the participants highlighted feeling that they had to partner with their body. They described how, although it is normally the ED that sets the rules, the rules appeared to be different in nature, where they were bound by the outdoor conditions. As Eva explained, “you have to find a place to sleep, you have to find food, you need shelter from the wind, it’s like, things like this, it is more like the basic needs in life…” In nature, she continues, “there are sort of different life rules than in the rest of life.”

Interviewer: Yes… how is that?

Eva: It’s quite good actually, and it feels kind of right in a way, or maybe, well, you know you have to pay attention to, when it’s cold you need to dress up, if you are tired you need to take a break. That you play together with your body a little bit more, too, and a bit more, like, learn to interpret it, and sort of acknowledge that if I am to have as good a hike as possible, I need to listen to it [she laughs], and that’s nice to learn and yeah…

As the rules were described as being different in nature, the participants commented that being in nature opened one up to self-care—meeting the basic needs of one’s body was essential for one to thrive in nature. Several of the participants related this to a shift from how their body appeared, to how it functioned.

Nora: Because, when you are out there, you feel how your body functions, it brings you where you are going, it’s like, there is no one commenting or no influence on how you are or who, how you look or which clothes you are wearing…

As Nora highlighted, and in accordance with several of the other participants, this focus on the body’s functioning rather than its appearance was described in connection to a contrast between being in nature and being in society, among other people. The unconditional requirements of nature were expressed as closely related to the experience of nature as being non-judgmental. This experience of being “just you and nature,” as Julie emphasized, seemed to be important—both for a shift in focus and to legitimate self-care.

## 4. Discussion

The objective of the current study was to explore embodying experiences with nature related to recovery in everyday life for persons experiencing EDs. The results will be discussed in relation to the literature on recovery, the phenomenology of the body, and embodying experiences with nature.

### 4.1. Recovery as Everyday Experiences and the Reciprocal Human–Nature Bond

An important objective of recent mental health recovery research has been to argue how recovery takes place within “normal” environments and activities [21]. As such, much of the recent recovery-oriented research supports the understanding that recovery is “not just an individual journey” [20]. Recovery has been highlighted as thorough relational processes with an emphasis on the connection between what takes place on the inside of a person and the socio-cultural situatedness of an individual [55]. Related to this, we seek to broaden the perspectives on *relational recovery* as not only appealing to an interconnectedness of human beings embedded in a social context [55], but also including the relationship between humans and nature.

Persons with experiences with EDs often highlight a feeling of distress as fundamental [14]. Hence, much of the effort in their ED recovery processes is related to alleviating some of this distress [34]. In keeping with this, the current study reported how nature was described as a less demanding environment that held several important calming qualities. These results are in accordance with prior research emphasizing nature’s calmness related to, for instance, the variation and accentuation of the senses, and open and free areas [56,57]. Moreover, albeit recognizing that quiet on the outside can still mean chaos on the inside, the experience of quietness related to natural sounds has been highlighted as having an important calming quality, both in the current study and in studies on nature and mental health more generally [56,57]. Thus, for the persons in the current study, what they related to as *nature* seemed to be closely attached to what they experienced in nature, in terms of presence, quietness, and calmness.

The connection between a desired balance between calmness on the outside and calmness on the inside could be argued to highlight the bond between humans and nature. In this, the body should not be seen as an object responding to stimuli in nature, but as an existential body, continuously experiencing and experienced, in movement and intentionally seeking out into the world [10,11]. The reciprocal “sense-full” interaction between our body and the world stresses the understanding that nature not only talks to us, but also listens [26]. The participants in the current study talked about how they could leave some of their worries in nature in a reciprocal exchange, in which they received some of nature’s calmness in return. Being in nature, then, could be seen as a dialogue between feelings and thoughts, and nature [58]. The experienced balance between one’s own feelings and the interpretation of nature’s expression (e.g., wind, rain, waves) has been highlighted by several studies [31,57,59], and supports the argument that a connection with nature holds the potential to enhance the relationship with one’s self [59,60].

The deep emotions that are related to the human–nature interaction could be understood as a sense of belonging, including a feeling of connectedness to a broader reality, and a sense of purpose and faith in a larger reality [6,57]. Persons, regardless of nationality and culture, seem to prefer natural environments with water features, large old trees, intact vegetation and minimal human influence [61]. Continuing this, and in accordance with the participants in the current study, Ottosson [59] accentuates the *timelessness* of features like rocks and large trees as fundamental in that it contributes to a change in perspectives related to one’s own situation. This understanding is supported by literature demonstrating that a focus on something larger than yourself is important for persons in ED recovery [17].

### 4.2. Experiences with Nature Challenge the Body–Mind Dichotomy

One of the most prominent results in the current study is the understanding of how the participants met nature through their body, particularly their feet. The varied terrain and the sense of the ground appeared to play a vital part in participants’ reconnecting with their own body. As highlighted by Sands [15], persons with EDs often experience their bodies as alien or separate. The results of the current study reveal the complexity and ambiguity related to the experiences with EDs as something primarily taking place in the mind, at the same time as the participants emphasized EDs as a difficult relationship with their own body. Related to this, the body–mind dichotomy seemed highly present in the participants’ stories. This could be understood as the persons themselves having been influenced by the Western way of thinking in dichotomies [58]. Moreover, the continued objectification of their body by several of the participants could also be understood in light of the fact that one of the main challenges related to EDs might be found precisely in the dialectic between having or being a body [62,63]. When the relationship with one’s own body is challenging or problematic, it might be perceived as easier to *have* a body rather than *be* a body. However, although using the dichotomy of mind and body as separate realms to reflect upon their understandings of EDs, the participants in the current study also emphasized the (re)connectedness of their body and mind, as it was precisely moving the body in nature that could help ease the distress in their mind. The sensory awareness embedded in interaction with nature has been emphasized as contributing to a shift in focus away from problematic thoughts and feelings [31]. This is in accordance with reports from the participants in the current study, where the “sense-full” intentionality of the outward perspectives helped put the inward perspectives to rest.

Discovering ways to forget oneself might be recognized as a common strategy related to dealing with EDs [62]. However, as highlighted by Duesund and Skårderud [62] (p. 59), the aim is that one’s body is “positively absent from [one’s] attention.” Related to this, the participants in the current study emphasized their experiences with nature as not only offering valuable breaks from troubling thoughts, but also including external orientations and attempts to restore their reciprocal contact with nature and the world. As emphasized by Merleau-Ponty [10], the inextricable link between body and life-world in a circular alternation between the subjective and objective implies that how nature is experienced is influenced by what one brings with them into nature. As reported by the participants in the current study, it was not necessarily the case that spending time in nature erased all their struggles. As such, and as they illustrated, ED recovery is perhaps best understood as long lasting processes. Being in nature, then, could be seen as a way to live with and deal with the experiences one has [21,64].

The complexity and ambiguity of experiences with EDs are related to an acceptance of oneself in the present as difficult [65], at the same time that the experience of nature as being unconditional and all-accepting is emphasized [57]. As revealed in the present study, nature appeared to be providing participants with a different set of “life rules.” Several studies have emphasized nature as being an environment without prejudice as important for opening up towards a reconnection with one’s own body [7,56,57]. Moreover, to be “cut-off from the body” has been associated with depriving a person from a vital source of self-care [15] (p. 35).

As argued by Crossley [9], it might have been the case that Descartes’ separation of mind and body was an attempt to save the human self-image from the objectification and desire of mechanical explanations of humans following the advancement of the natural sciences in the 17th century. While in the Cartesian tradition, the mind has priority over the body, Duesund [58] questions whether the body–mind dichotomy has again arisen—but this time, in reverse order, in which an instrumentalization of the body in today’s society leaves no room for the soul. This could be argued to be especially pertinent, as the objectification of the body through the ED at the expense of subjective experiences may inhibit the circulation between body and life [62]. It accentuates the ambiguity that persons with EDs are often working hard to avoid experiencing in their body, at the same time as the ED keeps them in an all-consuming relation with their body [14]. Several of the participants in the current study emphasized a shift in their focus on appearance to a focus on bodily function, related to the non-judgmental atmosphere in nature. While this function-orientation could be understood as gratefulness for having a body capable of functioning in nature [66], it may also run the risk of becoming a new instrumentalization of the body.

### 4.3. Strengths and Limitations

There are several strengths and limitations of the current study that should be noted. First, women were strongly overrepresented in the sample, which seems to be common in studies on EDs in general [67], as well as on EDs and nature [27,31]. A possible explanation for the fact that few men responded with interest during our recruitment process might be that EDs have long been associated with women, and men might still find it difficult to recognize themselves in the ED terminology [67,68].

Second, the current study presents an in-depth inquiry of experiences with nature of eight persons in Norway, and should thus be considered according to this context. Transferring the results to other (particularly non-Nordic) cultures—both in terms of access to nature as well as the position nature holds in Nordic countries (practically, socially, and cultural-ideologically) [69,70]—might represent a potential challenge. Moreover, previous research has argued that it is useful to distinguish between the features of “wild” nature versus other types of nature [71]. With this in mind, it might be seen as a limitation that the current study presents a variety of Norwegian nature-landscapes according to the participants’ definition of “nature in their everyday life.” In addition, the different studies discussed in relation to the current research project also include a variety of natural environments.

However, with this article’s objective in mind, we believe a dynamic approach towards nature could be argued as a strength when exploring lived experiences of everyday life. While recognizing the importance of both therapy in nature and the therapeutic benefits of nature [27], one could perhaps argue that there is an important difference between using a designed and fenced-in therapy garden, where the natural environment is integrated into existing exercises [31] and meeting nature “on its own terms” [56]. That we risk losing something valuable in our connection with nature when nature as a place for “healing” becomes organized and institutionalized has been argued for many decades [72]. Rather than a dose-response relationship with nature [73] or a static understanding of how humans respond to different types of nature (i.e., stimuli), the current study argues that the benefits of interactions with nature are well embedded in a dynamic and reciprocal relationship. In this relationship, we as humans are influenced by nature, which again is influenced by what we bring with us in nature [12,47].

Further, it is important to emphasize that the current research project does not aspire to investigate the potential “effects” of interaction with nature, but merely seeks to explore how persons with EDs experience nature. It is outside the scope of this study to examine if experiences with nature involve a more or less beneficial strategy for persons experiencing ED compared to other health conditions. However, this might constitute a potential avenue for future research. In this study, persons who defined themselves within the inclusion criteria specified in the announcements of the study contacted the first author. Although it was deemed important that the initiative to partake in the research project came from the participants themselves, this could also be argued to be prone to selection bias, as we only know the experiences from those who were able to reach the first author. In addition, it might be worth commenting that not all persons find meaning, are comfortable, or prefer spending time in nature, although this was not the focus of this study.

The hermeneutic phenomenological approach chosen means that the authors constitute both the strengths and the limitations of a study, and require a self-critical reflexivity towards their own backgrounds, beliefs, attitudes, and knowledge throughout the entire research process [40]. That the first author is an outdoor education teacher, is herself fond of nature, was born and raised in Norway, and spends a substantial amount of time in Norwegian nature arguably influenced her critical lens. However, this background could also be argued as a strength in her aim to maintain a focus on the person as a citizen, one just like everybody else, in a cultural context where *friluftsliv* is normative for a substantial amount of the population [74].

### 4.4. Implications for Practice and Future Research

The current study holds several implications with regard to both future research and practice. First, understanding persons’ experiences with EDs recovery as encompassing broader health concerns, wellbeing, and quality of life offers insight into understanding those experiencing EDs as persons in their everyday contexts, not only defined by their symptoms or diagnosis [24]. The current study illustrates how spending time in environments experienced as nurturing for their recovery represented a crucial strategy for dealing with their ongoing situation. Future studies may therefore benefit from integrating a focus on ED recovery that takes place in both treatment and everyday life. Methodologically, as most research on nature and mental health is arguably still based on comparative studies, dose-response relationships and questionnaires [5], there is also a need for research aiming to explore lived experiences with nature in persons’ everyday life.

Second, although keeping in mind the importance of professional help, exclusively focusing on EDs from clinical perspectives may undermine the resources of the person and inhibit important perspectives on recovery [34]. Moreover, it is relevant to note that a substantial number of persons with EDs never receive treatment from health care professionals [75]. There is thus a need for further exploration of lived experiences with ED recovery from first-person perspectives [17,33,34].

Third, the current study regards the body as essential in EDs, and highlights the need for further emphasis on embodying experiences as essential in both ED treatment specifically and ED recovery processes more generally. Although an increasing amount of research supports interdisciplinary cooperation in ED recovery approaches [63,76], much of the literature still concerns more conventional treatment of BN and BED, such as cognitive–behavioral and psychosocial approaches, at times supported by psychopharmacologic treatment [77]. We acknowledge that what is found helpful in recovery processes varies; the aim is not to abandon therapeutic or clinical approaches, but to argue the benefits of more holistic perspectives towards ED recovery processes.

Fourth and lastly, this study’s exploration of experiences with nature aspires to contribute to the growing understandings of the human–nature interaction in health research more generally, and the emphasis of nature in both therapy and in everyday life. While several studies thus far have delineated the psychological, physiological, spiritual, and social benefits for humans [5,6], future studies may also benefit from a focus on the dynamic and reciprocal human–nature interaction.

## 5. Conclusions

The results from this study illustrate how embodying experiences with nature allow for a (re)connection with one’s own body. The peacefulness and calmness of nature, together with an engagement of the senses, were expressed by participants as providing room for them to come as they were, with all of their experiences. Moreover, the study revealed experiences of being with nature as allowing self-care, as nature required something of them—albeit in an unconditional and non-judgmental way. Their embodying engagement with nature, particularly through sensing the ground with their feet, highlighted the complexity and ambiguity of EDs, as it facilitated a re-connection of body and mind while simultaneously emphasizing the advantages of focusing on something outside themselves (e.g., the ground, or nature more generally). Related to this, and supported by our results, we argue for the importance of an integrative focus on body and mind in ED recovery. Moreover, the results emphasize ED recovery as long lasting processes that take place within multiple aspects of a person’s everyday life. Closely following this is the argument that understanding experiences with nature as representing valuable breaks from the distress associated with difficult situations offers valuable perspectives on the reciprocal bonds between humans and nature.
